# Individual Differences in Working Memory Capacity Modulates Semantic Negative Priming from Single Prime Words

**DOI:** 10.3389/fpsyg.2016.01286

**Published:** 2016-08-29

**Authors:** Juan J. Ortells, Carmen Noguera, Dolores Álvarez, Encarna Carmona, George Houghton

**Affiliations:** ^1^Department of Psychology, University of AlmeríaAlmería, Spain; ^2^School of Psychology, University of Wales BangorBangor, UK

**Keywords:** working memory capacity, negative priming, individual differences, attentional control, semantic priming

## Abstract

The present study investigated whether *semantic* negative priming from single prime words depends on the availability of cognitive control resources. Participants with high vs. low working memory capacity (as assessed by their performance in complex span and attentional control tasks) were instructed to either attend to or ignore a briefly presented single prime word that was followed by either a semantically related or unrelated target word on which participants made a lexical decision. Individual differences in working memory capacity (WMC) mainly affected the processing of the ignored primes, but not the processing of the attended primes: While the latter produced reliable positive semantic priming for both high- and low-WMC participants, the former gave rise to reliable semantic negative priming only for high WMC participants, with low WMC participants showing the opposite positive priming effect. The present results extend previous findings in demonstrating that (a) single negative priming can reliably generalize to semantic associates of the prime words, and (b) a differential availability of cognitive control resources can reliably modulate the negative priming effect at a *semantic* level of representation.

## Introduction

Selection of a relevant stimulus from among competing irrelevant stimuli is a core cognitive ability. Without efficient selection, coherent interaction with a dynamic and complex environment becomes impossible. An influential paradigm in cognitive psychology that was originally developed to measure attentional selection is that of Negative Priming (NP). NP is typically observed in selective attention tasks that present target stimuli among distractors in two consecutive displays (the first called the prime display and the second called the probe display). The NP effect (Tipper, 1985) is the finding that observers are slower to respond to a target stimulus that appeared as a distractor stimulus on the prime display compared with a target stimulus that did not appear on the prime display (Dalrymple-Alford and Budayr, 1966; Tipper, 1985).

The NP effect has been observed for a variety of stimuli and a variety of populations (for reviews, see, e.g., Fox, 1995; Tipper, 2001; Frings et al., 2015). Unlike some other selection tasks (e.g., Stroop, flanker tasks), the NP task allows investigation of the fate of the representation of previously encountered but ignored stimuli. NP is commonly observed when the to-be-ignored distractor is “selected against” a concurrent attended target. However, further work has also reported reliable NP even in the absence of distractor stimuli on the prime display (i.e., single-NP; e.g., Milliken et al., 1998; Frings and Wentura, 2005; Noguera et al., 2007, 2015; Chao and Yeh, 2008).

There are at least two competing explanations for the NP effect. On the one hand, it has been taken to imply that successful selective attention to a target stimulus depends in part on selective inhibition of any distractors present. This forward-acting inhibitory account of NP is based on two fundamental ideas: First, both relevant and irrelevant stimuli are initially processed in parallel. Second, selection is a dual process in which an excitatory mechanism acting to enhance the processing of targeted information is complemented by an inhibitory mechanism acting to suppress (and/or decouple from potential effectors) the activation levels of the distractor’s internal representations. The residual inhibition associated with the distracting item is presumed to produce the delayed responding (NP) to this stimulus when it appears as a target on a subsequent probe display (Houghton et al., 1996). On the other hand, NP may reflect a backward-acting process of episodic retrieval (e.g., Fox and de Fockert, 1998). On this account, NP occurs when the current target triggers retrieval of a previous encounter with the same stimulus, which on that occasion served as an irrelevant distractor, causing a delay in the response selection process (e.g., Neill et al., 1992). Evidence has accumulated on both sides of this debate, and has led to some compelling hybrid models proposing how both forward-acting inhibition and backward-acting retrieval processes could contribute to the effect (e.g., Kane et al., 1997; Tipper, 2001). Yet, irrespective of this debate, there is converging evidence to suggest that the processes that contribute to NP are effortful and resource demanding. Thus, the NP effect depends critically on the availability of cognitive control (working memory) resources, which serve to minimize the processing of distractor information (e.g., Lavie et al., 2004; de Fockert et al., 2010; de Fockert, 2013).

Indirect evidence for this position is provided by studies on cognitive aging, which tend to show that elderly participants are disproportionally impaired compared to younger participants at tasks that require active rejection of distracting information. For example, by assessing Stroop interference and NP concurrently in the same procedure, Mayas et al. (2012) found that relative to younger participants, older adults showed not only an increased Stroop interference, but also reduced NP from irrelevant (distracting) stimuli, indicating that they failed to inhibit them. Note, however, that such evidence for a link between cognitive control resources and selective attention is mostly indirect, as a reduction in working memory capacity (WMC) in the older (vs. younger) groups is often assumed rather than directly measured in these studies.

More direct evidence that cognitive control functions are necessary for NP to occur comes from research showing that NP is directly modulated by working memory load and/or WMC (for recent reviews see Redick et al., 2007; de Fockert, 2013). By measuring distractor interference in a context of varying working memory load (e.g., high vs. low mental load), several studies have demonstrated that a to-be-ignored distractor gives rise to reliable NP only under conditions of low memory load. Under high memory load, NP is eliminated or is even converted to positive priming (PP; e.g., Engle et al., 1995; Chao and Yeh, 2008; de Fockert et al., 2010; see also Chao, 2011). Other studies seek correlations between WM span and selective attention by comparing the performance of low vs. high WM span groups. WM capacity is typically measured with a complex span task, such as the Operation Span -Ospan- task (e.g., Unsworth et al., 2005). In the Ospan task, participants perform number calculations while adding to a list of words (or letters) they keep in memory; working memory span is the sum of all correctly recalled word (or letter) lists. Consistent individual differences in a NP task as a function of WM capacity have been reported, such that only high WM capacity participants showed reliable NP, whereas low WM capacity participants did not (e.g., Conway et al., 1999; see also Long and Prat, 2002). Together, these findings suggest that having low WM capacity has the same effects on NP as having a high load on working memory.

It should be noted, however, that research so far examining a dependence of NP on cognitive control (working memory) resources, has used different versions of the *identity* NP paradigm, in which the prime stimulus itself is *repeated* as the target stimulus on the subsequent probe display. It thus remains unclear whether a differential availability of control resources (i.e., participants with high vs. low working memory capacities) could modulate NP not only at a relatively low feature (perceptual) level, but also at a more abstract (*semantic*) level of representation. This issue was addressed in the present research.

Traditionally, a central issue in research on the NP effect concerns the level of representation at which it operates (Damian, 2000; see also Tipper, 1985). The reason for that interest is that NP has usually been considered a relevant finding not only in promoting dual conceptions of selective attention (i.e., excitatory mechanisms would be complemented by inhibitory processes), but also in suggesting that an ignored stimulus may undergo a deep level of processing, as proposed for example by late-selection attention models (e.g., Deutsch and Deutsch, 1963). Consequently, it would be critically important to demonstrate that NP does not depend on the physical identity between the prime and the target, and it can also generalize between semantic associates belonging to the same semantic category (cf. Neill and Mathis, 1998). In fact, Tipper (1985) and Tipper and Driver (1988) demonstrated that NP could generalize to the semantic associates of the prime stimulus (e.g., *cat*–*dog*). They explained this *semantic* NP effect by appealing to a *spreading prospective inhibition* mechanism that mirrors *automatic spreading activation* suggested to underlie semantic PP from attended stimuli (e.g., Collins and Loftus, 1975; Neely, 1977). According to Tipper and Driver, when a prime distractor is ignored, inhibition beginning at the central representation of that stimulus would spread to related representations, lowering their activation levels below baseline (see Houghton and Tipper, 1994, for a different explanation of semantic NP in terms of inhibitory processes in selective attention). Note that semantic NP can also be accounted for by *episodic retrieval* theories (Neill et al., 1992; Neill, 1997) by assuming that during prime selection “not-respond” (or “to-be-ignored”) tags can be placed not only on the distractor stimulus itself, but also on related items activated by the distractor. As suggested by Neill (1997), the current target stimulus would cue the retrieval of past processing episodes involving similar stimuli. Accordingly, when the retrieved episode includes information about the response and/or relevance of that stimulus, semantic NP would occur if a previously encoded item semantically related to the current target had been ignored.

Although it is usually accepted that NP can also rely on the semantic similarity between the prime distractor and the probe target, the evidence of semantic NP has been elusive thus far, especially when words are used as prime stimuli (e.g., Tipper and Driver, 1988; see Fox, 1995, for a review). Unlike the NP effects from identity or spatial tasks, semantic NP effects from prime words have often been weak and difficult to replicate, with the observed effects being highly sensitive to minor procedural/methodological differences, such as the strength -and forward vs. backward direction- with which the prime and target words are associated; or the type of probe task.

Still another factor that might be critical in explaining differences in semantic NP effects across conditions and studies is the existence of individual differences in attention control and working memory capacities (WMC). As previously noted, it has not been investigated yet whether the semantic NP effect could critically depend on WMC. It remains possible that obtaining contradicting semantic NP findings even under highly similar task conditions could at least partly be due to differences between studies regarding the differential proportion of participants showing high vs. low WMC.

Note that in order to obtain a reliable semantic NP effect it is not only necessary that the prime distractor is actively ignored (as it is the case regarding identity NP). It is also critical that the activation of its abstract semantic memory representation *spreads* to the representations of semantically related items, and that inhibition (or “to-be-ignored” or “not-respond” tags) is also applied on these representations related to the ignored prime. It is well established that prefrontal areas, in particular the dorsolateral prefrontal cortex (PFC), seem to support not only attentional control and working memory functions (e.g., active manipulation of task-relevant information, and interference blocking of competing information), but it also plays a role in *semantic processing* (e.g., Petersen et al., 1988; Shivde and Thompson-Schill, 2004), particularly under task conditions that encourage a *strategic* or *controlled* processing of semantic information (e.g., high proportion of related prime-target pairs; a long prime-target stimulus onset asynchrony -SOA-; see for example, Hutchison, 2007).

Based on results of several priming studies in thought-disordered schizophrenic patients, Spitzer and colleagues have suggested that PFC could modulate not only controlled processing, but also automatic semantic processing (e.g., Spitzer et al., 1993; see also Kiefer et al., 2005). Schizophrenic patients frequently exhibited increased semantic priming effects for both directly (*hen–egg*) and indirectly (*lemon* [sour]*–sweet*) related prime–target word pairs, compared with healthy control subjects, particularly under conditions that minimize strategic processes (i.e., at short prime-target SOA; a low relatedness proportion –RP). According to these authors, PFC focuses retrieval of semantic information to meaning aspects related to the context, so that automatic spreading activation in semantic networks reaches only closely related nodes (e.g., *lemon– sour*). Because of dysfunctional prefrontal information processing in schizophrenic patients, spreading activation during semantic access would be stronger and far-reaching, thus resulting in increased direct and indirect automatic priming effects.

Because individuals differ greatly in their PFC functioning (Gazzaniga et al., 1998; Kane and Engle, 2002) and because this difference can underlie individual differences not only in attentional control and working memory tasks, but also in semantic priming tasks, it not implausible that semantic NP could be even more sensitive than identity NP to a differential availability of cognitive (working memory) resources. The present research addresses this issue.

### Current Study

As noted above, research examining semantic NP from words has usually produced smaller effects than those obtained from identical prime-probe stimuli, with reliable semantic NP being observed under some limited conditions. Furthermore, a sizeable portion of previous studies have reported a failure to replicate semantic NP from words (see Fox, 1995, for a review), such that some authors have questioned *whether semantic NP actually exists* (e.g., MacLeod et al., 2002). It is thus not surprising that semantic NP is only barely mentioned in the recent review by Frings et al. (2015; p. 1578).

Based on these considerations, it is important to establish the boundary conditions under which consistent semantic NP may be found. Over the last two decades, evidence has accumulated that obtaining reliable semantic NP from words critically depends on (i) instructing participants to actively ignore the prime distractor (e.g., Ortells and Tudela, 1996; Milliken et al., 1998); (ii) using a prime-probe SOA interval long enough to allow an efficient engagement of controlled attentional processes (e.g., Ortells et al., 2001; Noguera et al., 2007); (iii) presenting related prime–target pairs that are both categorically and associatively related (e.g., *cat–dog*; *nose–mouth*; see for example Abad et al., 2003), particularly in the forward direction (e.g., Hutchison, 2002); and (iv) requiring participants to make a relatively demanding forced-choice task, such as lexical decision or semantic categorization (instead of naming) on the probe target (e.g., Richards, 1999; Ortells et al., 2001; Hutchison, 2002; Noguera et al., 2007).

In the present research we used a paradigm (Daza et al., 2007; Noguera et al., 2007, 2015) that respects all the above conditions, in which participants are required to make a lexical decision (word/non-word) on a probe target that is centrally presented either alone or along with a letter-string distractor (depending on the experiment). The target display is preceded by a prime display containing a single central word (presented briefly and post-masked), which on related trials (50% of word trials) is associatively and categorically related to the upcoming target (e.g., *tiger–lion*; *face–eyes*). On unrelated trials (50% of word trials), the prime–target pairs are unrelated words belonging to different semantic categories (e.g., *tiger–face*; *eyes–lion*). Immediately before the prime display onset either YES (in green) or NO (in red) is presented, varying randomly from trial to trial. Participants are instructed that if they see YES they should “attend to and remember” the following (prime) word, as it would be tested in a subsequent memory task. However, if they see NO they should actively ignore the following prime word, as it would otherwise disrupt their memory for the “attend to and remember” words^1^. Previous work (Noguera et al., 2007, 2015) has shown a consistent interaction between attentional instructions and priming effects. The YES cue gave rise to reliable semantic PP, while the NO cue produced reliable semantic NP, regardless of whether the probe target was presented with or without distractors.

Apart from replicating this pattern of results, the main goal of the current work was to explore whether *semantic* NP from ignored words depends on the availability of cognitive control resources. Accordingly, participants with high vs. low working memory capacities (as assessed by their performance in several attentional control and complex span tasks) performed a semantic NP task similar to that used by Noguera et al. (2007; Experiment 4). To the extent that semantic NP depends on WMC, we expected to find a reliable interaction between ignored priming and WMC. Thus, the prime words that participants were instructed to actively ignore should produce reliable semantic NP only for high-WMC participants, with no NP effect or even an opposite PP effect being found for low-WMC participants.

By contrast, no reliable interaction of priming with WMC was expected regarding the attended prime words. Although our prime-target SOA (600 ms) seems to be long enough for participants to consciously generate likely targets, the prime-target relatedness proportion employed was 0.5, which is unlikely to recruit effortful strategic processes (Neely, 1977, 1991). In addition, the non-word trials may be considered unrelated trials, as a non-word target was always preceded by a prime word belonging to a different category. Hence the aggregate probability that a prime word is followed by a semantically related target is 0.33. In this case, the priming effects should mainly reflect automatic spreading activation in semantic networks, as participants could not reliably use the attended prime to develop expectancy for specific related targets during the interval between prime and target onset (e.g., Neely, 1991; Hutchison, 2007; Hutchison et al., 2014). To the extent that the attended priming effects in this task are mainly the result of automatic processing mechanisms (i.e., spreading activation), they should be insensitive to individual differences in WMC. We therefore expect that the attended primes should produce reliable PP effects of a similar size for both low-WMC and high-WMC participants (namely, no reliable interaction between attended priming and WMC).

## Materials and Methods

### Participants Screening for Working Memory Capacity and Attention Control

A sample of 200 native Spanish speakers with normal or corrected-to-normal vision were prescreened for WMC on the basis of their performance on an automated versions of operation and symmetry complex span tasks (see Unsworth et al., 2005, 2009 for more task details). These complex span tasks have demonstrated good reliability and validity, and are strongly predictive of a person’s complex cognition, fluid intelligence, or attention control abilities.

The automated operation span task (AOSPAN; Unsworth et al., 2005) requires participants to solve a series of simple mathematical operations while trying to remember a variable set of unrelated items. For example, the participant is shown an operation such as “(3 × 1) -1 = ” for a period of time; the next screen shows a digit such as 6, and the participant is to click on the “yes” or “no” box on the screen to indicate whether the digit is the correct answer to the arithmetic operation. The participant is then shown a letter for 800 ms. After three to seven such items, the participant views a matrix of letters on the screen, attempting to recall the letters in the order in which they were presented (by clicking them with mouse). Participants are encouraged to put equal emphasis on math performance and on letter recall. The number of operation-letter pairs per series varied from three to seven (three series of each length are performed, and the order of presentation was random, so that the participant could not predict series length). The dependent measure, or *global Aospan score*, is the sum of letters correctly recalled from series that are recalled perfectly (all letters in their correct order with no intrusions). The total possible score thus ranged from 0 to 75. If accuracy on the mathematical operations was below 85%, the subject was excluded. Subjects who scored 44 or higher were classified as high span and those who scored 24 or lower were classified as low span. These cutoffs reflect the upper and lower quartiles of our 200-subject pool.

The structure of the automated symmetry span task (ASYMSPAN; Unsworth et al., 2009) is similar to that of the AOSPAN just described, with the following exceptions. First, instead of remembering letters, subjects are presented with a 4 × 4 matrix of blank squares, with one square colored in red on a given trial. Second, instead of solving mathematical problems, subjects make vertical symmetry decisions about an 8 × 8 figure composed of black and white squares. At recall, the subject must indicate the location of the squares within the matrix that were colored for that trial in the same order as they appeared by clicking on the cells of an empty matrix. Finally, the list length can vary between two and five (with three trials of each list length), for a total of 42 symmetry figures and square locations on the task. The dependent measure, or *global Asymspan score*, is the number of square locations recalled in the correct sequential order (with no intrusions) across all trials. The total possible score thus ranged from 0 to 42. Subjects who scored either 23 or higher, or 12 or lower, were classified as high low span, respectively. These cutoffs reflect the upper and lower quartiles of our 200-subject pool.

In addition to obtaining independent global scores in the two span tasks for each participant, we also calculated a *z*-score WMC composite. Performance on each task was transformed into a *z*-score based on our database of over 200 scores. A *z*-score WMC composite was created by averaging across the two complex span tasks’ *z*-scores for each participant. Quartiles were then computed from the averaged distribution, with *z*-scores of +0.575 and -0.58 corresponding, respectively, to the upper and lower quartiles of our 200-subject pool.

The participants also performed a version of the antisaccade task (Hutchison, 2007; see also, Kane et al., 2001; Unsworth et al., 2004). Previous work with this task has demonstrated differences in groups thought to differ in WMC, such as older vs. younger adults, schizophrenics or patients with lesions in the PFC vs. healthy controls (e.g., de Jong, 2001; see Everling and Fischer, 1998, for a review). In this task, participants are told that an asterisk will appear to the left or right of fixation, and immediately followed by a target stimulus (*O* or *Q*). The latter appears either on the same (prosaccade condition), or on the opposite side of the screen (antisaccade condition), as the asterisk. Participants were informed that their task in the antisaccade trials was to look away from the flashed asterisk in order to identify the target before it disappeared. Trials began with a white fixation (+) presented on a gray background for either 1000 or 2000 ms. Following the fixation, a white asterisk (^∗^) appeared 3° to the left or right of fixation for 300 ms. Both asterisk location (left or right) and asterisk delay (1000 or 2000 ms) varied randomly on a trial-by-trial basis to prevent participants from anticipating when or where the asterisk would appear. Following the asterisk, the target appeared 3° to either the same (prosaccade block) or the opposite side of fixation (antisaccade block) for 100 ms and was immediately replaced by a backward pattern mask (##). The pattern mask was displayed for 5,000 ms, during which time the participants were to press either the *Q* or the *O* key to indicate the identity of the target. The timing of the trials was designed such that if participants accidentally made a saccade toward (as opposed to away from) the asterisk, they would not have time to plan and execute another saccade to the opposite side of the screen and reach the target. Participants completed a total of 128 trials: 64 trials (16 practice) for the antisaccade block, and 64 trials for the prosaccade block, with the order of blocks being counterbalanced across participants.

### Participants

Twenty-four low (19 women) and twenty-four high (19 women) WMC participants, who had scored respectively in the lower (<24) and upper (>44) quartiles in the AOSPAN task of our 200-subject database, were recruited for the lexical decision study. The *z*-score WMC composite for each participant in the high-WMC and low-WMC groups fell also within the upper (>+0.575) and lower (<-0.58) quartiles compared to our database (see **Table 1** below). Participants were between 18 and 51 years old (*M* = 21.9, *SD* = 6.7 for lows; *M* = 21.9, *SD* = 4.8 for highs), and all of them received credit toward course requirements as compensation. All participants signed a written consent after the nature and the consequences of the experiment had been explained. The experiment was conducted in accordance with the Declaration of Helsinki.

**Table 1:** Summary statistics for performance in the complex span (Aospan, Asymspan, and *z*-score composite) and attentional control (antisaccade and prosaccade trials) tasks by Low-WMC and High-WMC groups.

	Low-WMC	Group	High-WMC Group	
		
	*M*	*SD*	*M*	*SD*
Complex Span tasks				
Aospan score	15.38	6.53	52.83	7.68
Asymspan score	7.13	4.50	23.67	8.30
*z*-score composite	-1.10	0.31	+1.01	0.33
Antisaccade task				
Antisaccade trials				
RT (ms)	726	165	573	149
AC (%)	0.77	0.12	0.91	0.12
Prosaccade trials				
RT (ms)	535	157	471	106
AC (%)	0.96	0.062	0.096	0.098


### Stimuli and Apparatus

The stimulus set was similar to that used by Noguera et al. (2007; see also Noguera et al., 2015). It consisted of 48 concrete and familiar Spanish nouns of 4–7 letters length (16 per category) belonging to four semantic categories (geographical features and atmospheric phenomena, foods, animals, and body parts; see Appendix A), which were selected from the intra-categorical associative norms published by Callejas et al. (2003). From that 48-word set, 24 items were presented only as primes and the remaining 24 were presented only as targets (a different word set was presented during practice trials). A further set of 24-words (six from each category) of length 4–7 letters was selected, and one letter from each word was changed to produce an orthographically regular, pronounceable non-word.

**FIGURE 1:**
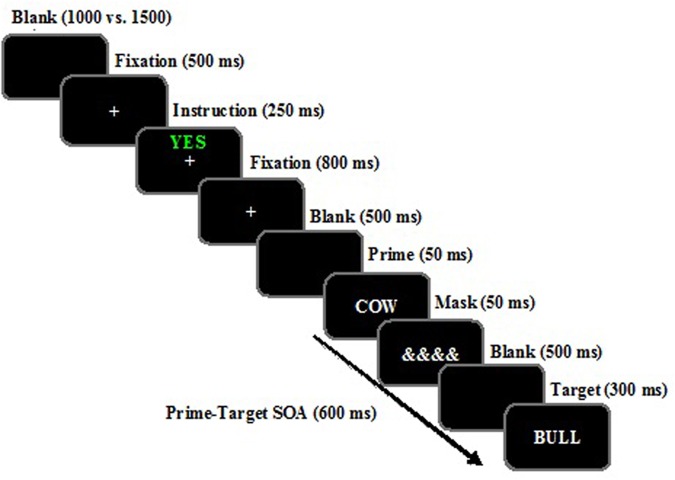
**Example of the temporal sequence of events when the target is a “word” presented for the instruction of Attending to and remembering (“YES” printed in green) the prime word.** The word stimuli shown here for related and unrelated trials have been translated from Spanish to English. Stimuli are not drawn to scale.

All stimuli were presented on a computer screen at a viewing distance of approximately 60 cm. Stimulus delivery and response recordings were controlled by E-prime software (Psychology Software Tools Inc.^2^). Each trial consisted of a sequence of nine critical displays (see **Figure 1**): Blank screen, fixation, instruction, fixation, blank screen, prime, mask, blank screen, and target. The fixation display consisted of a white asterisk (^∗^) presented at the center of the screen on a dark gray background. The instruction display consisted of either the word “YES” printed in green or the word “NO” printed in red and presented just above fixation. Both the prime and the probe displays consisted of a single uppercase letter string (4, 5, 6, or 7 letters) presented at the center of the screen, and subtending an average visual angle of about 2.21° wide and 0.49° high. The mask display consisted of a series of ampersands (“&&&&&&”) at the center of the screen. Participants responded by pressing either the “m” or “c” keys, indicating whether the target letter string was either a meaningful word or a non-word. Mappings of word/non-word decisions and correct key (m or c) were counterbalanced across participants.

### Design and Procedure

General task instructions were displayed on the monitor and also orally delivered. The timing of the events was as follows (see **Figure 1**): (1) Blank screen presented for a random duration between 1000 and 1500 ms; (2) Fixation display (^∗^), presented for 500 ms; (3) Instruction display, consisting of either the word “SI” (yes) in green or “NO” (no) in red at the center of the screen for 250 ms. The participants were instructed that if they saw “YES” they should “attend to and remember” the following word as it would be tested in a subsequent memory task. However, if they saw “NO” then they should “ignore” the following prime word, as it was a distractor that would disrupt their memory for the “attend to and remember” words. The attentional cue varied randomly from trial to trial; (4) Fixation display (^∗^) presented for 800ms; (5) Blank screen for 800 ms; (6) Prime display containing a single word displayed at fixation for 50 ms; (7) Masking display presented for 50 ms; (8) Blank screen of 500 ms (thus resulting in a prime–target stimulus onset asynchrony – SOA – of 600 ms); (9) Probe display containing a single target letter string for 300 ms, on which the participants made a lexical decision (word vs. non-word). The participants were told to press the appropriate response key (m or c) as quickly and accurately as possible, with the computer emitting a 500-ms beep if the participants made an error.

Participants took part in a single session (lasting about 25 min) consisting of 24 practice trials (16 word and 8 non-word trials) followed by 144 experimental trials (divided into 2 consecutive blocks of 72 trials each), consisting of 48 trials containing a non-word target and 96 trials containing a word target. All the 48 non-word trials could be viewed as “*unrelated*” trials, because every non-word target was preceded by a prime word belonging to a different category to that from which the non-word had been created^3^. Of the 96 word trials, the prime and target words belonged to different categories on 48 (unrelated) trials, and they were highly associated category members on 48 (related) trials. On half of both the 48-related and the 48-unrelated word trials, participants were instructed to “attend to and remember” the prime word preceding the target (attended trials), whereas on the other half they were instructed to actively “ignore” the prime word preceding the target (ignored trials). Different prime words were always presented on both “attended” and “ignored” trials, with the prime words assigned to each instruction type being counterbalanced across participants. For each participant, every prime word (e.g., LION) appeared two times on *non-word trials* (i.e., the prime word was followed by two different unrelated non-words), and four times (either as a to-be-attended or as a to-be-ignored prime) on *word trials*: Twice being followed by a highly associated target word from the same semantic category (i.e., the first ranked exemplar on forward direction in the norms of Callejas et al., 2003; e.g., LION-TIGER), and twice by an unrelated target word belonging to a different category (e.g., LION-HAND).

The main factors manipulated in the experiment were WMC, manipulated between-participants at two levels (High vs. Low capacity), Instructions (Attend to vs. Ignore), and Prime-Target Relatedness (Related vs. Unrelated). The last two factors were manipulated within-participants with a different random order for each individual. Half of the trials were “Attend to and remember” and half were “Ignore.” Within each of these conditions, half of the target words were related to the preceding prime words and the remaining half was unrelated. The breakdown of the trials was the same for the non-word targets but only the trials containing word targets were analyzed.

Participants were informed that after completing the experiment, they would carry out a recognition task about the previously attended words. Although participants were told that there was only one recognition test, two tests were actually presented, one about the attended and the other about the ignored items. They had to classify each word as “new” or “old” depending on whether or not the word had appeared in the experiment. A total of 48 words were presented: 24 “old” words presented as either attended or ignored words on the prime display, and 24 “new” items (not previously presented) belonging to the same four semantic categories mentioned above. Each participant received a different random ordering of the old and new items.

## Results and Discussion

### Working Memory (Complex Span) and Attention Control (Antisaccade) Tasks

Descriptive statistics (means and standard deviations) for performance in both the two complex span tasks (global span and *z*-composite scores), and the antisaccade task (RTs and accuracy in the prosaccade and antisaccade blocks) for both high-WMC and low-WMC groups are presented in **Table 1.**

As can be seen in **Table 1**, high-WMC individuals performed significantly better than low-WMC individuals in the two complex span tasks [Aospan: *t*(46) = 18.1; Asymspan: *t*(46) = 9.6; *z*-score composite: *t*(46) = 17.6; all *p_s_<* 0.001]. High-WMC participants also reliably outperformed low-span participants in both response latencies [*t*(46) = -3.34, *p* = 0.002] and accuracy [*t*(46) = 4.52, *p <* 0.001] for the antisaccade trials. In contrast, high- and low-WMC participants performed virtually identically in the prosaccade trials where fast and accurate target identification would be aided by a relatively automatic orienting response. Thus, the differential performance of the two WMC groups does not simply reflect a generalized performance deficit in low- compared with high-WMC subjects, such as lack of attention or slower processing-speed.

These impressions were confirmed by results of further mixed analyses of variance in which WMC (high vs. low) was treated as a between-participants factor, and saccade type (antisaccade vs. prosaccade trials) as the within-participants variable. The ANOVAs yielded reliable main effects of both WMC and saccade type, and more interestingly, a reliable interaction between these two variables in both RTs [*F*(1,46) = 9.25, *p <* 0.001, η^2^ = 0.18] and Accuracy [*F*(1,46) = 17.9, *p <* 0.001, η^2^ = 0.28] in the antisaccade condition. The analyses of these interactions demonstrated that high- and low-WMC individuals showed a fairly similar performance on the relatively automatic prosaccade trials. In clear contrast, they reliably differed in the antisaccade trials, with low-span participants having significantly longer latencies [*t*(46) = 3.34, *p* = 0.002] and reduced accuracy [*t*(46) = 4.52, *p <* 0.001] compared to high-span participants.

The pattern of inter-individual task performance differences was also assessed by a correlation analysis for the entire sample of 48 subjects. Verbal and visuospatial span tasks were highly correlated (*r* = 0.78, *p* < 0.001), suggesting that individual differences in WMC reflect predominantly domain-general rather domain-specific resources. In addition, high-WMC was correlated with a better performance in the antisaccade trials for both response latencies [Aospan: *r* = -0.37, *p* = 0.009; Asymspan: *r* = -0.51, *p* < 0.001; *z*-composite: *r* = -0.46, *p* < 0.001], and accuracy [Aospan: *r* = 0.49; Asymspan: *r* = 0.63; *z*-composite: *r* = 0.59, all *ps* < 0.001]. In contrast, WCM measures did not reliably predict either latency or accuracy in the prosaccade trials^4^.

### Priming Task

Trials containing an incorrect response (2.4% of trials) or those with reaction times (RTs) falling more than 2.5 standard deviations from the overall mean RT (2.1% of trials) were removed from analyses. Mean RTs from correct word trial responses were calculated for the participants in each WM capacity group (High vs. Low) as a function of Instructions (Attend to vs. Ignore), and Prime-Target Relatedness (Related vs. Unrelated). Two analyses of variance (ANOVA) were conducted, one with the participants (*F*_1_) and other with words (*F*_2_) as the random factor. Mean RTs and mean error rates as a function of WM Capacity, Instructions and Relatedness are shown in **Table 2.**

**Table 2:** Mean (SD) reaction times (*in ms*), and error percentages (in %) as a function of Working Memory Capacity (High vs. Low capacity), Instructions (Attend to vs. Ignore the prime), and Prime-target Relatedness (Related vs. Unrelated).

	Instructions	
	
	Attend to	Ignore
Low-WMC Group		
Unrelated	515 (108.1)	510 (153.7)
	3.5 (0.06)	4.9 (0.07)
Related	459 (122.5)	474 (164.2)
	1.6 (0.03)	2.1 (0.03)
High-WMC Group		
Unrelated	574 (211.2)	523 (162.3)
	1.7 (0.04)	2.4 (0.05)
Related	504 (215.1)	553 (183.1)
	1.0 (0.01)	2.5 (0.05)


The error rate analysis revealed only a significant main effect of Relatedness [Related = 1.6%; Unrelated = 3.3%; (*F_1_*(1,46) = 9.71, *p* = 0.003, η^2^ = 0.17; *F_2_*(1,46) = 5.39, *p* = 0.025, η^2^ = 0.11].

**FIGURE 2:**
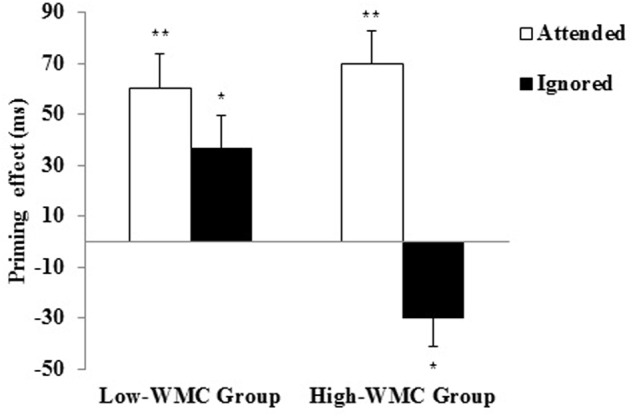
**Semantic priming effects (unrelated minus related) for Attended and Ignored primes for High-WMC and Low-WMC participants.** The vertical lines depict the standard error of priming scores for each condition. Significant contrasts are highlighted by asterisks (^∗^*p* < 0.05; ^∗∗^*p* < 0.01).

The analysis of RTs showed a significant main effect for Relatedness [Related = 498 ms; Unrelated = 531 ms; *F_1_*(1,46) = 22.98, *p <* 0.001, η^2^ = 0.23; *F_2_*(1,46) = 14.34, *p <* 0.001, η^2^ = 0.24], which interacted with Instructions [*F_1_*(1,46) = 25.53, *p <* 0.001, η^2^ = 0.36; *F_2_*(1,46) = 12.63, *p <* 0.001, η^2^ = 0.22]. More interestingly, there was a reliable three-way interaction between Instructions, Relatedness, and WMC [*F_1_*(1,46) = 11.54, *p* = 0.001, η^2^ = 0.201; *F_2_*(1,46) = 7.01, *p* = 0.011, η^2^ = 0.13]. Further analyses of this interaction revealed a differential priming pattern from attended vs. ignored primes as a function of WMC (see **Figure 2**).

The attended prime gave rise to reliable PP effects for both High-WMC [+70 ms; *F_1_*(1,23) = 28.97, *p <* 0.001, η^2^ = 0.56; *F_2_*(1,23) = 16.13, *p <* 0.001, η^2^ = 0.41] and Low-WMC participants [+50 ms; *F_1_*(1,23) = 16.5, *p <* 0.001, η^2^ = 0.42; *F_2_*(1,23) = 11.72, *p* = 0.002, η^2^ = 0.34]. This pattern was as predicted. With a relatedness proportion below 0.50, the semantic priming effects would be mainly driven by automatic spreading activation, rather than controlled expectancy generation, regardless of whether a short or longer prime-target SOA is used (Neely, 1991; Kiefer et al., 2005; Hutchison et al., 2014). This view is supported by the absence of a reliable correlation between the attended priming effects and any WMC measure (Aospan, Asymspan, *z*-composite).

Regarding the ignored primes, a differential (opposite) priming pattern as a function of WMC was found, as revealed by a reliable interaction between Ignored Priming and WMC [*F_1_*(1,46) = 11.54, *p* = 0.001, η^2^ = 0.201; *F_2_*(1,46) = 9.89, *p* = 0.003, η^2^ = 0.18]. Further analyses of this interaction revealed that the ignored primes produced reliable PP in Low-WMC individuals [+36 ms; *F_1_*(1,23) = 7.95, *p* = 0.01, η^2^ = 0.26; *F_2_*(1,23) = 5.54, *p* = 0.027, η^2^ = 0.19], but an opposite NP effect in High-WMC individuals [-30 ms; *F_1_*(1,23) = 6.69, *p* = 0.017, η^2^ = 0.225; *F_2_*(1,23) = 4.42, *p* = 0.047, η^2^ = 0.16]. Given the finding of 36 ms *facilitation* from ignored primes in the low WMC group, we directly compared this to the 50 ms facilitation from the attended primes in the same group, and found it not to reach significance [*t*(23) = 1.36, *p* > 0.19]. Thus the low WMC group showed only an unreliable reduction in positive semantic priming following the ignore cue, compared to the very reliable semantic NP shown by the high WMC group.

Further evidence in support of a dependence of semantic NP on WMC is the finding that the ignored priming effects (unlike the attended priming effects) did reliably correlate with all the WMC measures in our study [Aospan: *r* = -0.40, *p* = 0.005; Asymspan: *r* = -0.41, *p* = 0.004; *z*-composite: *r* = -0.33, *p* = 0.02]. These results replicate and extend some previous findings (e.g., Engle et al., 1995; Conway et al., 1999; Chao and Yeh, 2008; de Fockert et al., 2010) in showing that a differential availability of cognitive control (WM) resources reliably modulates NP not only at the feature (perceptual) level but also at a more abstract (*semantic*) level of representation.

An inspection of **Table 2** shows that whereas in the low WMC group RTs to unrelated targets were fairly similar for attend and ignore conditions, the same did not occur in the high WMC group. In this latter case, response latencies to unrelated targets in the ignore condition were reliably slower [*F*(1,23) = 5.8, *p* = 0.025, η^2^ = 0.19] than those in the attend condition. One could argue that this differential response pattern in the unrelated condition could be at least partially responsible for the reliable priming by instructions interaction that was found in the High-WMC group. While the source these differences remains unclear, it is very likely they are mainly due to non-systematic (random) between-participants variability. Note that in some prior single-prime NP studies (e.g., Ortells et al., 2003; Noguera et al., 2007; see also Daza et al., 2007) a fairly similar interaction between Instruction and Relatedness (and ignored NP effects of a similar size across experiments) is found regardless of whether RTs to unrelated targets are either different or fairly similar between attend and ignore instructions (see for example Ortells et al., 2003; Experiments 1 vs. 2; see also Noguera et al., 2007; Experiments 2 vs. 5). Second and more importantly, a re-examination of our data in the High-WMC group revealed that in 3 of the 24 participants RTs to the unrelated targets were much slower for the attended than for the ignored condition (2 of the 3 showed NP effects from the ignored primes). We then conducted further ANOVAs in which the data from these three participants were removed. The results of these new analyses again revealed a reliable three-way interaction between Instructions, Relatedness, and WMC [*F_1_*(1,43) = 10.7, *p* = 0.002, η^2^ = 0.20; *F_2_*(1,46) = 5.97, *p* = 0.018, η^2^ = 0.12]. Even more importantly, in the High-WMC group the Instructions by Relatedness interaction was again significant [*F_1_*(1,20) = 29.64, *p* = 0.001, η^2^ = 0.201; *F_2_*(1,23) = 17.65, *p* < 0.001, η^2^ = 0.43], with the attended prime producing reliable PP [+60 ms; *F_1_*(1,20) = 19.9, *p <* 0.001, η^2^ = 0.50; *F_2_*(1,23) = 7.82, *p* = 0.010, η^2^ = 0.25] and the ignored primes reliable NP [-36 ms; *F_1_*(1,20) = 8.8, *p* = 0.008, η^2^ = 0.31; *F_2_*(1,23) = 4.43, *p* = 0.046, η^2^ = 0.16]. But interestingly, whereas RT differences between instruction types for related targets were very similar to those found with 24 participants [Attend = 477 ms; Ignore = 551 ms; *F_1_*(1,20) = 17.93, *p <* 0.001, η^2^ = 0.47; *F_2_*(1,23) = 10.34, *p* = 0.004, η^2^ = 0.31], the RT differences between attend and ignore instructions for the unrelated targets now failed to reach significance [Attend = 537 ms; Ignore = 515 ms; *F_1_*(1,20) = 1.9, *p >* 0.18; *F_2_* < 1]. In addition, the correlation analysis for the reduced sample of 45 participants again showed that the ignored priming effects (but not the attended priming effects) did reliably correlate with all the WMC measures [Aospan: *r* = -0.50, Asymspan: *r* = -0.48; *z*-composite: *r* = -0.51, all *ps <* 0.001]. On this basis we would argue that the reliable interaction between Instructions and Relatedness does mainly depend on differences in WMC.

On the other hand, note that whereas Low-WMC individuals were overall faster, but less accurate in the priming task (RT = 489 ms; error rate = 3.2) than the high-WMC group (RT = 539 ms; error rate 1.7), the main effect of WMC was not statistically significant either in RTs or in error rates analyses. These results provide further evidence that general processing speed is not the mechanism responsible for the differential performance of the two WMC groups in our study.

### Prime Recognition Task

The recognition scores for both the attended and ignored primes deviated significantly (*p* < 0.05) from zero. The analysis of recognition scores showed a reliable main effect for Instructions [*F*(1,46) = 44.9, *p <* 0.001, η^2^ = 0.49], such that they were higher for the attended (*d*′ = 3.16) than for the ignored primes (*d*′ = 1.78), thus providing further evidence that the attentional instructions did indeed influence the processing of the primes. The main effect of WM Capacity was also significant [*F*(1,46) = 5.82, *p* = 0.02, η^2^ = 0.12], such that the recognition scores were reliably higher for High-WMC (*d*′ = 2.94) than for Low-WMC participants (*d*′ = 2.011). Moreover, the interaction between Instructions and WM Capacity was also significant [*F*(1,46) = 4.39, *p* = 0.043, η^2^ = 0.043]. This was due to the fact that the differences between high- and low-WMC participants on prime recognition was significant for the attended [High-WMC = 3.84; Low-WMC = 2.48; *t*(46) = 3.68, *p* = 0.001], but not for the ignored primes [High-WMC = 2.04; Low-WMC = 1.54; *t*(46) = 1.01, *p >* 0.31]. Consistent with the above findings are the results of the correlation analysis, which showed that the recognition scores for only the attended primes reliably correlated with all the WMC span scores [Aospan: *r* = 0.43, *p* = 0.002; Asymspan: *r* = 0.41, *p* = 0.004; *z*-composite: *r* = 0.44, *p* = 0.02]. These results suggests that a higher WMC was associated with an increased recognition of primes words that participants were instructed to attend and remember.

On the other hand, despite the reliable interaction between Instructions and WM Capacity, we also found that both high-WMC and low-WMC individuals performed reliably better for the attended than for the ignored primes [High-WMC: Attended = 3.84; Ignored = 2.04; *t*(46) = 5.5, *p <* 0.001; Low-WMC: Attended = 2.48; Ignored = 1.54; *t*(46) = 3.8, *p* = 0.001]. This is a relevant finding in demonstrating that the fairly similar PP effects from the attended and ignored primes that were observed in the low-WMC group, cannot be explained in terms of a lesser ability to follow the different instructions and/or to switch attention between them during the priming task.

## General Discussion

The current study had two main goals. The first goal was to replicate the differential semantic priming pattern (positive vs. negative) that was found by Noguera et al. (2007, 2015). As noted in the Introduction, the evidence of semantic NP from prime words has been elusive thus far, such that some authors have questioned the *existence* of the effect (e.g., MacLeod et al., 2002; see also Frings et al., 2015). Therefore, we consider it important to demonstrate that a consistent semantic NP from words can be found as long as several boundary conditions are fulfilled. The second and more important goal was to investigate whether *semantic* NP from single prime words could critically depend on the availability of cognitive control (working memory) resources. To this end, individuals high and low in WMC (as assessed by their performance in complex span and attention control tasks) performed a semantic NP task similar to that of Noguera et al. (2007; Experiment 4). They were instructed to either attend to or ignore a briefly presented single prime word that was followed by either a semantically related or unrelated target word on which participants made a lexical decision task.

Given the 50% prime-target relatedness proportion on word trials, it is highly unlikely that participants could strategically use the attended prime to develop expectancy for a specific related target word (Neely, 1991; Hutchison, 2007; Hutchison et al., 2014). Consequently, we predicted that the attended priming effects would not greatly affected by individual differences in WMC (i.e., no reliable interaction between attended priming and WMC). In contrast, to the extent that semantic NP depends on WMC, we expected that the ignored primes would give rise to reliable NP only in high-WMC but not in low-WMC participants (i.e., a reliable interaction between ignored priming and WMC).

As predicted, we found that individual differences in WMC mainly affected the processing of the ignored (irrelevant) primes, but not the processing of the attended (relevant) primes: Whereas positive semantic priming was found in both participant groups, reliable semantic NP was shown only by the high WMC group. In stark contrast, the low WMC participants showed a PP effect from ignored primes. Indeed, this facilitatory effect was only slightly reduced compared to that generated by the attended primes, and the difference (14 ms.) was not significant. However, on the prime recognition task the low WMC group did show a reliable difference between attended and ignored primes, and overall, only the former condition showed a correlation with WMC. This supports the view that encoding of attended items into memory for a later recognition test is an active process which depends on WMC. At least for the low WMC group, ignoring an item for memory encoding appears to be more of a passive process, a decision not to engage the (effortful) encoding mechanism on a stimulus that is otherwise fully processed (leading to semantic facilitation). On the basis of the semantic NP results, the high WMC group appears to have the capacity to engage a more active, inhibitory, processing of to-be-ignored primes, suppressing the spread of activation through the semantic network.

As noted in the Introduction, the NP effect has been examined in great detail because it provides a window into the processes involved in selective attention. Note that the task requirement of selecting a target against distractors is retained in the standard NP procedure, in which participants are presented with couplets of prime and probe displays containing at least two stimuli, the to-be-responded target and the to-be-ignored distractor. It thus makes sense that attentional selection of a target against its distractor is a critical aspect in theories of NP, including the inhibition account (Tipper, 1985, 2001; Houghton and Tipper, 1994; Houghton et al., 1996) and the retrieval account (Neill et al., 1992; Neill, 1997; Neill and Mathis, 1998). According to the inhibition account of NP, the principal factor underlying NP is the suppression of the competing distractor by an inhibitory mechanism in the selection process of the prime display. According to the retrieval account, response to a probe target is hampered when its presentation retrieves a tag incompatible with the current behavioral goal, such as “do not respond”. In both accounts, selection in prime trials would lead to either the inhibition of the distractor or a tag associated with the distractor.

At first sight, obtaining NP from a single ignored prime (single-prime NP) would run counter to the traditionally accepted assumption that such an effect occurs because of a reaction to the distractor interference in selective attention situations. As pointed out by Milliken et al. (1998), because selection was not required during the presentation of a single prime stimulus (i.e., there was no target to which participants responded during prime presentation), the prime was not selected against, and hence not inhibited. It could, however, be argued that in an experimental procedure requiring participants to “ignore” the first (i.e., the prime) of the two stimuli presented in a rapid temporal sequence, the act of ignoring a single prime might involve the same inhibitory (or “do-not-respond” tagging) mechanisms that can actively suppress distracting items presented in irrelevant spatial locations on more conventional NP tasks. In this view, single-prime NP could also be explained in terms of selective attention processes, if the assumption is made that selective inhibition (or do-not-respond tagging) acting on pre-activated internal (abstract) representations of a prime word can operate not only under “spatial” co-ordinates (i.e., *where* to attend vs. ignore), but also under a “temporal” (i.e., *when* to attend vs. ignore) dimension (see Tipper, 2001, for a similar line of argument).

On the other hand, the dependence of NP effects on the presence of distractor stimuli in the probe display has been usually found only for tasks such as letter identification (e.g., Moore, 1994) or word (or color) naming (e.g., Milliken et al., 1998; Neill and Kahan, 1999). But when a (perhaps much more demanding) forced-choice binary task, such as a lexical decision or a semantic categorization is used on the probe trials, there are numerous previous reports of reliable NP even if probe distractors are absent (e.g., Yee, 1991; Ortells and Tudela, 1996; Richards, 1999; Ortells et al., 2001; Abad et al., 2003; Daza et al., 2007; Noguera et al., 2007, 2015).

While the current study was not mainly intended to investigate the underlying mechanism of NP, our findings of reliable semantic NP for high but not for low WMC individuals can be well accommodated by inhibitory accounts of NP, which assume that forward-acting attentional inhibition is resource demanding. Thus, a lower WMC could be associated to a lesser ability of otherwise-engaged control mechanisms to effectively inhibit and reject the processing of the to-be- ignored primes, thus explaining the lack of NP in the low-WMC group. This latter finding does not necessarily imply an absence of inhibition (or attention control) abilities in low-WMC individuals. Recent working memory research on normal aging (e.g., Gazzaley et al., 2008; Jost et al., 2011) have reported evidence that the selective deficit in suppressing task-irrelevant information during working memory encoding in older adults, would be *slowed* or *delayed* rather than generally impaired. Whether low-WMC individuals could show semantic NP from ignored primes (or even perform equivalently to high-WMC subjects) if a longer prime-target SOA had been used in our task remains an interesting issue for future research.

The dependence of semantic NP on WMC, as well as the reliable correlation between WMC scores and the ignored priming effects that we observed in the present research would also be consistent with both the inhibition (Hasher et al., 1999, 2007) and the executive attention (Engle and Kane, 2004) theories of working memory. The former account states that older adults and individuals low in WMC primarily have impairment in the ability to reduce (inhibit) interference from task-irrelevant information. The second account assumes that individual differences in WMC would mainly reflect variation in a domain-general attention control ability, needed to actively maintain task relevant representations in the face of distraction (e.g., sustain the task goal and constrain the focus of attention to relevant target items). Our findings that the ignored primes produced semantic NP only in the high-WMC group could thus be attributed to their better ability to either inhibit the ignored (irrelevant) information (Hasher et al., 2007), or maintain task relevant information in an active state and to block and inhibit irrelevant representations from gaining access to WM. There are some recent demonstrations that individuals high in WMC also exhibit improved performance on several memory tasks requiring controlled search abilities, such a free recall, cued recall, or item recognition when recollection and not familiarity is needed. Unsworth and Engle (2007), Unsworth and Spillers (2010) have proposed a dual-component (maintenance/retrieval) model, which can be viewed as an outgrowth of the executive attention account. According to this view, WMC would be composed of both attention control abilities (e.g., active maintenance in the face of distraction) and secondary memory abilities (controlled search), which are necessary to transfer information in and out of working memory. Although our prime recognition task mainly aimed to check the effectiveness of our instruction manipulation, the finding that the attended (and to be remembered) primes were better recognized by high-WMC than by low-WMC participants would also be consistent with this maintenance/retrieval theory.

Further evidence in support of both inhibitory and attention control theories of WMC is provided by the results in the antisaccade task. In our study, both verbal and visuospatial span scores showed reliable correlations with response latency and accuracy in the antisaccade, but not in the prosaccade trials. In fact, high- and low-span individuals showed a fairly similar performance on the prosaccade trials, but not on the antisaccade trials, with the low-span group showing longer latencies (and a lesser accuracy) than the high-span group (see **Table 1**). This pattern replicates that obtained in some previous studies, at least under experimental conditions that make minimal demands on cognitive control, as is the case when the prosaccade and the antisaccade trials are presented across different blocks (Kane et al., 2001; Unsworth et al., 2004; the present study). Under these conditions, it has been suggested that individual differences in working memory span would mainly reflect differences in suppression (inhibition) of prepotent responses (reflexive saccade), rather than in directing the focus of attention (looking forward the flashing cue), which is thought to rely on an exogenous, automatic attentional-capture that does not require the recruitment of executive control. Note also that the absence of significant differences in overall reaction times between the two WMC groups for both the antisaccade trials and the priming task, clearly demonstrates that the differential performance of the two WMC groups in our study cannot be attributed to a generalized performance deficit in low- compared with high-WMC subjects, such as slower responding.

Whereas the findings of reliable semantic NP for high-WMC but not for low-WMC individuals are consistent with inhibitory (and attention control) accounts, they are more difficult to explain by a strict episodic retrieval account of NP. To the extent that the retrieval of prior episodic traces, which contributes to the NP effect, is usually assumed to be automatic (Logan, 1988), it remains unclear why a differential WMC should modulate an automatic backward-acting retrieval process. It could be argued that differences in the availability of cognitive control resources might affect the processes involved in labeling or tagging the prime stimulus as more or less relevant. If a lower WMC resulted in the ignored prime being less clearly labeled as irrelevant on the prime display, then retrieval of episodic traces associated with these primes would be less likely to interfere with responses to the probe target, and thus these primes would produce less NP (or even PP) compared with ignored primes that were more successfully labeled as to-be-rejected distractors by participants showing higher working memory capacities. Note, however, that if low-WMC individuals were less able to mark the to-be-ignored primes as irrelevant, then they should also produce recognition scores for these stimuli that are fairly similar to those for the attended primes. But this was clearly not the case in the present research. We found instead that the recognition of ignored primes was reliably impaired for both high- and low-span participants. This latter finding also argues against the possibility that low-WMC participants had more difficulty than high-WMC participants to understand and/or follow the different attention instructions (attend vs. ignore) in our study.

The distinction between forward-acting attentional inhibition and backward-acting episodic retrieval theories of NP can be viewed as being somewhat similar to the distinction between proactive and reactive control, which was originally proposed by Braver and colleagues to account for impaired cognitive control exhibited in schizophrenia patients and older adults (e.g., Braver et al., 2001). Proactive control involves maintaining goal information in an accessible state so as to direct attention toward goal-relevant stimuli and away from potential internal and external distractions. This form of cognitive control is effortful and preparatory in nature, as uses predictive cues to prepare for a response to a specific upcoming target. In contrast, reactive control is a backward-acting process that is automatically triggered by target onset and involves retrieving prior contextual (e.g., goal) information from long-term memory. In contrast to proactive control, the reactive form of control does not require continuous effort or monitoring of the environment, but instead involves using a target stimulus to retrieve appropriate actions from long-term memory.

The task frequently used to assess this dual-control model is the AX-Continuous Performance Test (AX-CPT), which requires a response to a specific probe target (X letter) only if it follows a specific cue (A letter), and to withhold responses otherwise (e.g., an X letter preceded by a B letter). AX targets occur on 70% of all letter sequences, so a strong expectancy to make a target response is created when the letter A is presented. Proactive control in this paradigm involves maintaining the A cue in working memory and using it to maintain an expectancy to make an X response to the following stimulus. In contrast, reactive control involves using the probe (X) to retrieve the preceding cue from memory and is demonstrated by longer reaction times (or increased errors) in rejecting X targets that were preceded by other cues. Across numerous studies, Braver et al. have provided evidence for reduced proactive control among older adults and individuals with schizophrenia, relative to healthy young adults (see Braver et al., 2007; Braver, 2012, for reviews). Some recent AX-CPT studies have also demonstrated that healthy young adults high in WMC are more likely to efficiently use proactive strategies than low-WMC individuals (e.g., Redick and Engle, 2011; Hutchison et al., 2014; Redick, 2014).

The assumption that individuals vary greatly in their ability to maintain context (e.g., task instructions, previous cues) to guide future behavior, would be similar to the role that goal maintenance plays in the executive-attention theory by Engle and Kane (2004). One could argue that the high- and low-WMC participants in our study would differ in their ability to represent and maintain the different instructions (attend vs. ignore) within working memory. The lack of NP in the low-WMC group could be attributed to a greater difficulty in these individuals to effectively represent and/or continuously update the attention instructions in working memory until the prime appeared. While we cannot rule out that individual differences in WMC might reflect a differential use of proactive vs. reactive control strategies, several observations are pertinent here. First, as above noted, the finding that the low-WMC group showed reliably better recognition for the attended than for the ignored primes indicates that they were able to maintain the attend/ignore instructions in working memory. Second, even assuming that high-WMC individuals make a more efficient use of proactive strategies, it remains unclear why this should result in negative, instead of reduced positive, priming from the ignored (irrelevant) primes in our task. Unlike the inhibitory accounts of NP (e.g., Houghton and Tipper, 1994; Tipper, 2001; see also Hasher et al., 2007), the context-processing view is noncommittal on whether a to-be-ignored single-prime word should produce either NP, or reduced PP, relative to a to-be-attended prime.

It is now widely agreed that working memory should be viewed as a multifaceted construct, as multiple mechanisms seem to be needed to explain individual differences in WMC (e.g., Shipstead et al., 2012, 2015; Unsworth et al., 2015). It has been recently suggested that variation in WMC and the relation between WMC and higher-order cognitive functions, reflects not only variation in domain-general attention control abilities, or variation in the ability to strategically retrieve information from secondary memory, but also variation in the size (or scope of attention) or capacity of primary memory.

By using WM tasks, such as change-detection/location (or visual arrays), which provide a relatively pure measure of storage capacity in a short-term buffer, a variety of recent studies have reported evidence that both behavioral (i.e., the k-index; see Cowan et al., 2005) and electrophysiological (i.e., Contralateral Delay Activity-CDA- amplitude; Vogel and Machizawa, 2004) estimates of a person’s storage capacity, do reliably correlate with complex span performance and also with measures of broad cognitive functions (e.g., fluid intelligence, or gF; see for example Cowan et al., 2005; Fukuda et al., 2010; Shipstead et al., 2014, 2015; Unsworth et al., 2015). Despite such evidence, it is unclear why individuals with larger storage capacities should also be more likely to show NP than individuals with smaller storage abilities. Some recent work suggests that visual arrays performance, just as complex span measures, is not strictly driven by a limited-capacity storage system, but it may also rely on controlled attention abilities (Cowan et al., 2006; Fukuda and Vogel, 2011; Shipstead et al., 2015; Unsworth et al., 2015). Whereas the relationship between attention control and storage capacity remains a lively debated issue (see for example the different conclusions reached by Shipstead et al., 2012, vs. Shipstead et al., 2014, 2015), for future research addressing the dependence of semantic NP on working memory resources, we recommend the use of visual array tasks as estimates of WMC. This would allow more directly investigate whether individual differences in primary memory storage capacity could result critical to obtain semantic NP.

## Conclusion

As predicted, we found that individual differences in WMC mainly affected the processing of the ignored (irrelevant) primes, but not the processing of the attended (relevant) primes: Whereas positive semantic priming was found in both participant groups, reliable semantic NP was shown only by the high WMC group. In stark contrast, the low WMC participants showed a PP effect from ignored primes. Indeed, this facilitatory effect was only slightly reduced compared to that generated by the attended primes, and the difference (14 ms.) was not significant. However, on the prime recognition task the low WMC group did show a reliable difference between attended and ignored primes, and overall, only the former condition showed a correlation with WMC. This supports the view that encoding of attended items into memory for a later recognition test is an active process which depends on WMC. At least for the low WMC group, ignoring an item for memory encoding appears to be more of a passive process, a decision not to engage the (effortful) encoding mechanism on a stimulus that is otherwise fully processed (leading to semantic facilitation). On the basis of the semantic NP results, the high WMC group appears to have the capacity to engage a more active, inhibitory, processing of to-be-ignored primes, suppressing the spread of activation through the semantic network.

## Author Contributions

All the authors contributed equally to:

(1) The conception and design of the work as well as analysis and interpretation of data

(2) Drafting the work or revising it critically for important intellectual content

(3) Final approval of the version to be published and

(4) All of them are agreed to be accountable for all aspects of the work in ensuring that questions related to the accuracy or integrity of any part of the work are appropriately investigated and resolved.

## Conflict of Interest Statement

The authors declare that the research was conducted in the absence of any commercial or financial relationships that could be construed as a potential conflict of interest. The reviewer RD and the handling Editor declared their shared affiliation, and the handling Editor states that the process nevertheless met the standards of a fair and objective review.

## Footnotes

1 While other NP studies have examined the impact of attentional instructions on prime processing, the different instructions have been associated with either different blocks or groups (e.g., Ortells and Tudela, 1996; Milliken et al., 1998). Under these conditions, a differential priming pattern for the attended and ignored primes might reflect some strategic difference between participants other than the effects of the instructions *per se*. To avoid this possible confound, the instruction either to attend or to ignore the single prime varied randomly from trial to trial (Noguera et al., 2007, 2015).

2 www.pstnet.com/eprime

3 We presented fewer non-word (48) than word trials (96) in order to reduce the “*non-word ratio*” (i.e., the ratio of trials on which a target is a non-word given that the target is unrelated to its word prime), which would in turn minimize the involvement of *retrospective semantic-matching* strategies in our priming task (see Neely, 1991 for a more detailed discussion on this issue; see also Ortells et al., 2003; Noguera et al., 2007, for a similar task procedure). Thus, given an unrelated prime-target trial (48 word trials + 48 non-word trials = 96 trials of the total), there was the same probability for the target to be either a “non-word” (48/96) or a “word” (48/96).

4 Several researchers have recently recommended the use of the partial instead of the absolute (global)-scoring method to analyze performance on complex span tasks (e.g., Redick et al., 2012). A *partial* score reflects the sum of items recalled in the correct serial position, regardless of whether an entire trial was recalled correctly. It has been suggested that relative to absolute-scoring, the partial-scoring method shows higher test-retest correlations, higher internal consistencies and correlations among the different complex span task. Accordingly, *partial* scores in ospan and symmetry span tasks we also computed for each participant. The overall result pattern was very similar to that found with *absolute* or global span scores. Namely, high-WMC participants performed significantly better than low-WMC individuals in both span tasks [Aospan: *t*(46) = 11.5; Asymspan: *t*(46) = 10; *z*-partial composite: *t*(46) = 14.7; all *ps <* 0.001]. The partial span scores of the two tasks were again highly correlated among them (*r* = 0.79, *p* < 0.001). All the partial WMC scores reliably correlated with performance in the antisaccade condition for both response latencies [Aospan: *r* = -0.35, *p* = 0.002; Asymspan: *r* = -0.49, *p* < 0.001; *z*-partial composite: *r* = -0.44, *p* = 0.002], and accuracy [Aospan: *r* = 0.50; Asymspan: *r* = 0.61; *z*-partial composite: *r* = 0.60, all *ps* < 0.001]. In contrast, no reliable correlation was found between any of partial WCM scores and either response latency or accuracy in the prosaccade block. Given that most prior work addressing the relationship between WMC and selective attention tasks has relied on absolute-scoring scores, all analyses reported in the current paper are based on the absolute scoring rather than on partial scoring method in order to remain consistent with prior work.
