# Supplementary material for: Individual Differences in Working Memory Capacity Modulates Semantic Negative Priming from Single Prime Words
Source: Front Psychol. 2016 Aug 29;7:1286. doi: 10.3389/fpsyg.2016.01286 (PMC5002416; doi:10.3389/fpsyg.2016.01286)
Supplement: Supplementary file 1 [file Data_Sheet_1.DOCX]

**Appendix A**

Pairs of related stimuli (and their English Translations) used in the Experiment. Associative strength (%AS) scores (in forward direction) from norms by Callejas et al. (2003) are also presented.

_____________________________________________________________________

### Primes Targets %AS

______________________________________________________________________

CABRA (GOAT) oveja (sheep) 73.4

PERRO (DOG) gato (cat) 89.6

LEON (LION) tigre (tiger) 84.4

SAPO (TOAD) rana (frog) 96.2

TORO (BULL) vaca (cow) 92.3

ASNO (DONKEY) burro (sawhorse) 85.4

CODO (ELBOW) brazo (arm) 66.7

MANO (HAND) dedos (finger) 58.3

MUELA (MOLAR) diente (tooth) 66.4

PELO (HAIR) cabeza (head) 60.0

LABIOS (lips) boca (mouth) 70.7

OIDO (EAR) oreja (lug) 74.1

CALOR (HEAT) frío (cold) 58.6

COSTA (COAST) playa (beach) 58.5

CRATER (CRATER) volcán (volcano) 65.9

GOLFO (GULF) cabo (cape) 44.3

GRUTA (GROTTO) cueva (cave) 57.3

RAYO (RAY) trueno (thunder) 39.1

AJOS (GARLIC) cebolla (onion) 74.7

MELON (MELON) sandía (watermelon) 85.5

NATA (CREAM) fresa (strawberry) 47.2

PAELLA (PAELLA) arroz (rice) 83.3

PERA (PEAR) manzana (apple) 70.6

ZUMO (JUICED) naranja (orange) 47.4
